# The Evidence for Diet as a Treatment in Migraine—A Review

**DOI:** 10.3390/nu16193415

**Published:** 2024-10-09

**Authors:** Kattia Valentine Nguyen, Henrik Winther Schytz

**Affiliations:** 1 Danish Headache Center, Department of Neurology, Copenhagen University Hospital—Rigshospitalet-Glostrup, 2600 Copenhagen, Denmark; 2 Department of Clinical Medicine, Faculty of Health and Medical Sciences, University of Copenhagen, 2200 Copenhagen, Denmark

**Keywords:** migraine, diet, food, clinical symptoms

## Abstract

**Background/objectives:** The connection between diet and migraine has gained increasing attention in migraine research due to its potential relevance as part of migraine treatment. This study reviewed the current evidence on the use of diets or specific foods in the prevention of migraine. **Methods:** A PubMed search was performed with the keywords “diet and migraine” OR “brain-gut-axis and migraine”. One author (KVN) screened titles, abstracts, and full-text articles and excluded or included them based on eligibility criteria. **Results:** A ketogenic diet and a “Dietary Approaches to Stop Hypertension” diet reduced attack duration (*p* < 0.002), frequency (*p* < 0.05), and severity (*p* < 0.01). The ketogenic diet also reduced monthly medication intake (*p* ≤ 0.05). A low-fat vegan diet mixed with an elimination diet reduced the attack duration (*p* < 0.01), frequency (*p* < 0.05), severity (*p* < 0.0001), and percentage of medicated headaches (*p* < 0.001). Elimination diet reduced attack duration (*p* < 0.05), frequency (*p* < 0.02), severity (*p* < 0.01), and medication intake (*p* < 0.002). Elimination diet with IgG-positive foods reduced attack frequency (*p* < 0.001), and total medication intake (*p* < 0.01). Gluten-free diet reduced frequency (*p* = 0.02) and severity (*p* = 0.013). **Conclusions:** Certain diets and food items may trigger attacks in some migraine patients, though the overall evidence supporting this is limited. Modifying a diet may reduce symptoms such as attack duration, frequency, severity, and medication intake. However, the included studies’ small populations and diverse study designs make the results difficult to apply in clinical practise. Further high-quality, double-blinded, randomised controlled trials are necessary to confirm the association between diet and migraine.

## 1. Introduction

Migraine is a frequent primary headache disorder characterised by recurrent headaches with two major subtypes: migraine with aura and migraine without aura. It is characterised by intense, throbbing, one-sided headaches, which are aggravated by physical activity and accompanied by nausea and/or photo- and phonophobia [1]. Migraine is a very common global neurological disorder, with an increased incidence over the past few decades [2]. Additionally, it has been ranked as the third most prevalent disorder along with the third highest cause of disability for people under 50 years of age [3,4]. This highlights the need for improved treatment and overall management of migraine.

Migraine treatment is primarily aimed at treating the acute attack and, if needed, using preventive medication [5]. However, if predisposing and trigger factors can be correctly identified and subsequently avoided, headache might be treated without pharmacological interventions [5,6].

Several food items have already been suggested to trigger migraine attacks, e.g., chocolate, fruits, caffeine, and alcohol [7,8]. The same is observed with preventive diets such as low-fat diet, high-omega-1 and low-omega-6 fatty acid diets, ketogenic diet, “Dietary Approaches to Stop Hypertension” diet (DASH diet), and plant-based diet [9,10,11]. These dietary components and regimens have shown effects upon exposure or withdrawal, indicating a direct influence on migraine [9,10,11]. Research, however, suggests that chocolate cravings might not cause migraine attacks but instead, they may be a part of the premonitory phase that occurs before the headache itself [12]. In this phase, people may experience symptoms as “early warning signs” such as mood changes, fatigue, or hunger/food cravings, especially for sweets like chocolate [12]. Some foods may, therefore, potentially affect the frequency, duration, or intensity of migraine attacks, constituting a modifiable aspect that requires further attention.

The possible connection between diet and migraine attacks is currently unknown. The enteric nervous system (ENS) can potentially influence and communicate with the central nervous system (CNS) [13]. This communication, however, occurs through vagal and pelvic nerves and sympathetic pathways, with complex bidirectional networks of neural, immunologic, and hormonal pathways known as the brain–gut axis [13]. While the precise mechanisms and pathophysiology are still under study, evidence suggests that the ENS plays a role in aspects of affecting migraine through various neuroendocrine processes, which can be influenced by changes in diet [14]. Thus, clinical trials on diet interventions are of scientific and clinical interest.

In the current review, we therefore found it timely to investigate the underlying evidence for using a specific diet to alter migraine attack frequency, intensity, and duration.

## 2. Methods

### 2.1. Search Strategy

The database PubMed was literature-searched for studies reporting observational data on diets’ influence in migraine symptoms with the following keywords: “diet and migraine” OR “brain-gut axis and migraine”. No year or language restrictions were applied. The literature search was performed on 10 July 2023 and again on 11 March 2024. 

### 2.2. Study Selection

Using Covidence, articles were selected based on a two-step screening process. In the first step, one independent reviewer, KVN, screened each title and abstract. The reviewer then included or excluded articles based on the eligibility criteria outlined in Table 1.

Inclusion criteria were studies with adult patients undergoing any food, dietary, or nutritional interventions compared to a control group; outcomes with any food items or diets as triggering factors or the treatment/prevention of migraine; retrospective or prospective observational studies, randomised controlled trials (RCTs), and non-RCTs; articles written in the English language; and lastly, that the diagnosis of migraine was based on any edition of the International Classification of Headache Disorders (ICHD) [1]. 

As the first iteration of the ICHD (ICHD-I) was published in 1988, articles from before 1988 were automatically excluded. 

Exclusion criteria consisted of studies involving paediatric patients (≤18 years); interventions with supplements, vitamins, or any medication; the comparison of macronutrients, caloric intake, or weight/BMI; conference papers, guidelines, opinions, editorials, letters, case reports, book chapters, or comments; articles written in any other language than the English language; studies with less than 10 patients; if data cannot be extracted properly; and studies reporting results on overlapping cohorts.

**Figure 1:**
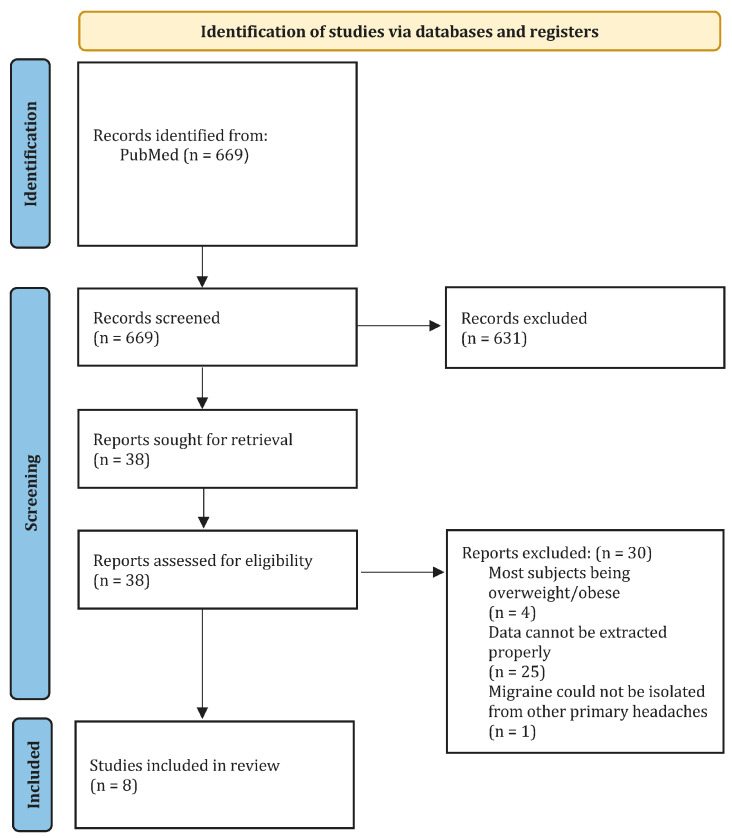
PRISMA flow chart diagram.

Articles passing the first screening were retrieved for a full-text screening and were reviewed for final eligibility in the second screening. The first and second screening was verified by HWS, and conflicts were resolved by HWS. The screening process is shown in Figure 1.

### 2.3. Data Extraction

Data were manually extracted from the articles by KVN. A data extraction sheet was used to extract data from each of the included studies that passed the second screening. In the end, the data were categorised into different diet-related interventional preventions. It was screened for the ICHD version and type of diet and included food items or groups, study design, sample size, gender, migraine type, and the used method/intervention. The results/effects of the clinical symptoms (duration, frequency, and intensity of attacks) before and after intervention and conclusions were also screened.

### 2.4. Quality of Assessment

To assess the quality of the eight included studies, one author, KVN, applied the Newcastle–Ottawa Quality Assessment Scale (NOS) for cohort studies, focusing on three main domains: the selection of study groups, comparability, and outcome assessment. Using this tool, the author evaluated the representativeness of the cohorts, the clarity in defining and controlling the diets, and whether confounding factors were managed. Additionally, the author considered the methods used to measure outcomes, the length of follow-up, and the consistency in monitoring participants, assigning stars accordingly. This was later confirmed by author HWS.

## 3. Results

The database search identified 669 records that were screened by title and abstract. Thirty-eight articles were then selected for a full-text screening. Of these, eight studies met the eligibility criteria and were included in the review, with seven focused on diet-related prevention and one outlining specific food item triggers. Two of the included studies were clinical crossover trials; one was a controlled trial, and one was a pilot study. The others did not directly address the study design. The study design and a narrative summary of the methods/interventions, results of interest, and conclusion are summarised and presented in Table S1.

### 3.1. Diet Interventions

#### 3.1.1. Ketogenic Diet

Bongiovanni et al. [15] and Lovati et al. [16] investigated the effects of a ketogenic diet (KD) on the characteristics of migraine attacks. Bongiovanni et al. [15] reported a 12-week KD reducing median attack duration (24 vs. 5.5 h, *p <* 0.002) and median frequency (30 vs. 7.5 headache days/month, *p <* 0.001). A decrease in the median number of drugs taken per month (30 vs. 6 doses, *p <* 0.003) was observed as well. For 83% of the 38 subjects, pain intensity was described as severe at baseline and improved to mild in 55% of patients (*p <* 0.003) [15].

Lovati et al. [16] conducted a pilot study with a 3-week KD. The first study showed a reduction in mean frequency (19.1 vs. 12.3 headache days/month, *p <* 0.05) compared to baseline. Additionally, a decrease in mean pain intensity (7.8 vs. 5.7 in NRS, *p <* 0.01) and mean monthly medication intake (24.9 vs. 11.5 doses, *p* = 0.05) was observed. In the second study, no difference in mean frequency was observed (14.5 vs. 10.5 headache days/month, *p* = 0.1). However, the mean frequency decreased (11.6 vs. 4.9 headache days/month, *p <* 0.001) among the responders [16].

#### 3.1.2. Low-Carb Diet

Lovati et al. [16] also investigated the effects of a low-carb diet (LCD, <40% of carbohydrates) in the same abovementioned study. None of the differences in mean frequency (21 vs. 18.7 headache days/month) and mean pain intensity (7 vs. 6.4 in NRS) were significant. The mean monthly medication intake (27.8 vs. 23.8 doses) did not show a significant difference either. 

There was no difference present between the KD and LCD groups in terms of mean headache frequency (*p =* 0.06). A difference was, however, present in terms of mean pain intensity (*p =* 0.02) and mean analgesic drug intake (*p =* 0.04) [16].

#### 3.1.3. DASH Diet

Another intervention conducted by Arab et al. [17] was a 12-week DASH diet compared to a control. They reported a reduction in mean attack duration (1.11 vs. 0.52 days, *p <* 0.001) and frequency (8.15 vs. 5.15 attacks/month, *p <* 0.001) for the DASH group. The DASH diet also reduced mean pain intensity (7.78 vs. 6.01 in VAS, *p <* 0.001). 

For the control group, mean attack duration (1.04 vs. 0.71 days, *p <* 0.01) and mean pain intensity (7.5 vs. 6.9 in VAS, *p <* 0.02) were reduced. The mean frequency (8.98 vs. 7.57 attacks/month, *p* = 0.113) was the only parameter that did not decrease in the control group.

A reduction in frequency (*p <* 0.03) and pain intensity (*p <* 0.001) between the DASH and control groups was observed. The duration tended to be lower in the DASH group than in the control group, but the difference was not significant (*p =* 0.053) [17].

#### 3.1.4. Elimination Diet

Several studies have also explored the outcomes of an elimination diet as an interventional prevention of migraine, including Alpay et al. [18], Bunner et al. [19], and Özön et al. [20].

Alpay et al. [18] randomised 30 patients to a diet that either included (provocation diet) or excluded (elimination diet) specific foods with high IgG antibody concentration. These included spices, seeds and nuts, seafood, vegetables, cheese, fruits, and sugar products.

Compared to the baseline (and provocation diet), they found a reduction in mean attack count (8.97 vs. 6.17 attacks, *p* < 0.001) after a 6-week elimination diet. The mean frequency (10.5 vs. 7.47 headache days/month, *p* < 0.001) and mean total medication intake (11.37 vs. 7.77 tablets, *p* < 0.01) were reduced as well. No difference was observed in mean attack duration (11.39 vs. 12.53h, *p* < 0.8) or mean headache intensity (6.02 vs. 6.07 on VAS, *p* < 0.5) [18].

Another randomised crossover study, conducted by Bunner et al. [19], reported a decrease in mean attack duration (6.1 vs. 4.8 h, *p* < 0.01) and mean frequency (2 vs. 1.7 headache days/week, *p* < 0.05). A reduction in mean pain intensity (6 vs. 3.6 in VAS, *p* < 0.0001) and mean headache intensity (4.3 vs. 3.1 in VAS, *p* < 0.001) was also observed. Finally, the study reported a decrease in the mean percentage of headaches requiring pain medication (65.2% vs. 41.3%, *p* < 0.001). These results were observed among completers during the 16-week diet period (a combination of a low-fat vegan diet and an elimination diet). Mean frequency was reduced (1.9 vs. 1.6 headache days/week and 2.1 vs. 1.8 attacks/week, *p* < 0.03) during the placebo supplement period as well [19].

Özön et al. [20,21] presented an association of an elimination diet with migraine headache parameters in two studies. In 2018, they conducted a 16-week elimination diet for two separate groups. The dietary restrictions were less restrictive for group 1 after the second month, but they were maintained in group 2. Already after 8 weeks, the mean attack duration reduced for both group 1 (29.44 vs. 22.2 h, *p* < 0.05) and group 2 (30.56 vs. 23.52 h, *p* < 0.04). The diet also reduced the mean frequency for group 1 (6.08 vs. 4.84 attacks/month, *p* < 0.02) and group 2 (5.96 vs. 4.68 attacks/month, *p* < 0.02). Finally, the mean pain intensity for group 1 (89.8 vs. 72.89 in VAS, *p* < 0.01) and group 2 (90.2 vs. 72.2 in VAS, *p* < 0.01) was reduced. After the 16-week elimination diet was completed, a difference was observed in mean attack duration (30.56 vs. 22.88 h, *p* < 0.03) in group 2. The same applies for mean frequency (5.96 vs. 4.64 attacks/month, *p* < 0.01) and mean pain intensity (90.2 vs. 71.4 in VAS, *p* < 0.01) in group 2. No significant difference was seen in group 1 [20].

In 2021, some of the same authors conducted an 8-week elimination diet specifically for elderly migraine patients over 65 years of age. Seventeen foods were determined as a trigger, of which all of them were excluded from the diets. These included wheat, egg, cheese, Nescafé, milk, chocolate, alcohol, sujuk, tea, red meat, onion, pickle, orange, oat, grape, garlic, and sesame. After the elimination diet, they reported a reduction in mean attack duration (32.65 vs. 18.74 h, *p* < 0.001) and mean attack count (5.74 vs. 4.16 attacks, *p* < 0.002). A decrease in mean pain intensity (82.26 vs. 62.26 in VAS, *p* < 0.001) was also reported. Lastly, the mean number of used analgesics (4.94 vs. 2.74, *p* < 0.001) and mean number of used triptans (1.65 vs. 0.87, *p* < 0.002) were reduced [21]. 

#### 3.1.5. Gluten-Free Diet

Ameghino et al. [22] analysed migraine patients with celiac disease and their response to an 8-week gluten-free diet (GFD). The study reported an improvement regarding frequency in participants with migraine without aura (MoA, improvement in 52,4%, *p* = 0.02) and migraine with aura (MWA, improvement in 46.1%, *p* = 0.02). Improvement was also seen regarding pain intensity in participants with MoA (improvement in 49.6%, *p* = 0.013) and MWA (improvement in 46.2%, *p* = 0.013). These parameters improved significantly more in patients with migraine than in those with tension-type headache (TTH) as well [22].

#### 3.1.6. NOS Scores

The NOS scores of each of the included studies are presented in Table 2. The study by Arab et al. [17] was by far assessed to be of the best quality, while most of the remaining studies ranged from 2 to 4 in the NOS score.

## 4. Discussion

The main finding of this review is that a few studies have shown that KD, DASH diet, low-fat vegan diet, and GFsD may decrease migraine parameters such as duration, frequency, and severity [15,16,17,19,22]. The monthly medication use was also found to be reduced in these diets [15,16,17,19,22]. An elimination diet of specific food items including IgG-positive foods may also decrease migraine frequency and monthly medication use [18,20,21]. Finally, an elimination diet and a low-fat vegan diet may decrease the attack duration and severity [19]. The mechanisms behind the possible effects of reducing migraine frequency and intensity will be discussed below.

### 4.1. Proposed Pathophysiological Mechanisms behind Diet Regimens

#### 4.1.1. Ketogenic Diet

In the ketogenic diet, carbohydrate restriction leads to energy consumption via the β-oxidation of fatty acids, resulting in the production of ketone bodies. Once the blood concentration of ketone bodies reaches 4 mmol/L, the ketone bodies become the main energy source for cells in the CNS [12]. This transition in energy source changes mitochondrial metabolism, and the glutamate/GABA ratio may reduce cerebral excitability, neuroinflammation, and the production of resistant oxygen species (ROS) [23,24,25]. 

#### 4.1.2. DASH Diet

The DASH diet is abundant in the cations K^+^, Mg^2+^, and Ca^2+^ and has low levels of Na^+^, which is known to play vital roles in brain function [26]. Unlike the other cations, elevated Na^+^ concentrations in both the blood and cerebrospinal fluid (CSF) have been observed in migraine attacks [26]. This suggests a potential involvement of Na^+^ in migraine pathophysiology. Alterations are therefore speculated to reduce the resting potential and action potential threshold in neurons. Additionally, Mg^2+^ is suggested to prevent migraines by blocking N-methyl-D-aspartate receptors, inhibiting serotonin-dependent vascular spasms, and prostacyclin-dependent vasodilation [27,28]. The diet itself also includes lots of fruits and vegetables that are high in anti-inflammatory and antioxidant components [29], known to modulate neuroinflammation associated with migraine [30]. 

Ca^2+^ from dairy has also been shown to inhibit the 1α-25-dihydroxycholecalciferol and angiotensin-converting enzyme (ACE), which may modulate inflammation [31]. 1α-25-dihydroxycholecalciferol was found to stimulate MCP-1 expression in 3T3-L1 adipocytes and inflammatory cytokine production from both adipocytes and macrophages [32]. Ca^2+^ may, therefore, indirectly be responsible for suppressing oxidative and inflammatory stress in adipose tissue [31].

#### 4.1.3. Low-Fat Vegan Diet

A low-fat vegan diet reduces oestrogen concentration and activity by increasing serum concentrations of sex-hormone binding globulin (SHBG), which inactivates oestrogens [33,34]. It can thereby indirectly affect migraine similar to the way oestrogen concentrations during menstrual cycles are strongly associated with migraines [35]. 

#### 4.1.4. Elimination Diet

Identifying provocative foods in elimination diets may rely on recognising associations between specific food items and triggered migraine attacks. While such subjective observations can guide dietary choices, they often lack insights into the underlying pathophysiology. This applies unless we were to isolate those food items, as demonstrated by Alpay et al. [18]. The risk with this approach is that it relies entirely on the migraine patient’s ability to recognise the association. It is, therefore, subject to both false positive and false negative attributions.

Inflammation and immune system activation play a role in migraine pathophysiology. Since the gut is a significant site of immune system activity, dietary factors can impact its responses throughout the body, including the CNS. Nitric oxide (NO) and the calcitonin gene-related peptide (CGRP) contribute to these responses. 

Except for IgG4, all IgG subclasses induce an inflammatory response when in contact with its respective (food) antigens [18]. Measuring IgG concentrations for various food items can effectively help identify suspected food item triggers, allowing for a more precise diet adjustment. 

Some dietary nutrients may have antioxidant and anti-inflammatory properties that help maintain redox homeostasis by activating antioxidant defence systems such as the Nrf2 pathway [36,37,38]. These nutrients follow the concept of hormesis, a biphasic dose–response process, where low/moderate doses are neuroprotective, inducing anti-inflammatory and analgesics effects, while high doses can be toxic, inhibiting these protective pathways [39]. This highlights the importance of evaluating doses carefully to avoid potential toxicity.

#### 4.1.5. Gluten-Free Diet

Similarly, the gluten-free diet reduces the intestinal inflammatory response and antibody levels, potentially improving nutrient absorption [22]. Patients with celiac disease have demonstrated strong IgA reactivity with blood vessel structures in the brain and substantial immunoinflammatory activation. This was found in their intestinal biopsy samples and peripheral blood cells characterised by a high chronic release of cytokines and other vasoactive molecules [40,41]. These molecules include IFN-γ and IL-2, -4, and -10 [40,41]. However, more evidence is needed to understand and explain the association fully.

Hence, so far, the abovementioned results suggest a possible value of a nutritional strategy for migraine treatment. RCTs involving larger samples examining diets and isolated food items or groups are, however, still highly needed. The RCTs are especially needed to investigate if the specific diets or restrictions may be beneficial in migraine treatment.

### 4.2. Limitations

There are several limitations to this review. Studies that explicitly reported the entire population or the majority of the population as overweight or obese were excluded. This is a limitation as it would have represented a more realistic migraine population, and excluding the studies would potentially skew the overall results. However, it lowers the chance of weight (loss) being a factor affecting the results, as being overweight/obese can increase the frequency and severity of migraines [42,43,44]. 

Another limitation of this review was that the studies included were conducted on various patient populations and had different study designs, which makes it impossible to assess which diet would be the most optimal for migraine patients. Bongiovanni et al. [15] included a population with medication overuse headache (MOH). Subjects with MOH may have distinct characteristics such as different medication histories, treatment responses, and treatment strategies, potentially leading to more favourable results regarding medication intake. Comparing their results to subjects without MOH may affect the overall findings, making it difficult to isolate the impact of MOH itself. 

Medication use was also allowed during all the interventions, but most studies did not provide detailed information about it. Ameghino et al. [22] did not address medication use. Alpay et al. [18] and Bunner et al. [19] did not specify which drugs were allowed. Bongiovanni et al. [15] and Lovati et al. [16] allowed patients to use analgesics. One study by Özön et al. allowed analgesics and triptans [21]; meanwhile, they did not specify what drugs were allowed in the other [20]. Arab et al. [17] reported patients taking beta-blockers, topiramate, tricyclic antidepressants (TCAs), tetracyclic antidepressants (TeCAs), serotonin–norepinephrine reuptake inhibitors (SNRIs), sodium valproate, triptans, gabapentin, and benzodiazepines. 

Generally, it can be complicated to isolate the impact of the dietary intervention if medication is allowed. Not specifying medication use can reduce the studies’ controllability, and variability in the types and dosages among participants can skew the results. Allowing medication, however, mimics real-life conditions where patients often take medications alongside dietary changes. This enables studies to assess whether diet can influence overall medication use and makes it more applicable to clinical settings. 

One of the studies by Özön et al. also involved an elderly population aged >65 years [21]. Thus, migraine characteristics and responses may be related to the specific age group, making it impossible to generalise the specific findings [45]. Furthermore, Arab et al. [17] included hypertensive patients and clearly stated that they did not assess women’s menstrual cycle status, which are variables that can influence migraine outcomes [1]. Generally, none of the studies addressed the influence of the menstrual cycle of female migraine patients during interventions. Not accounting for these variables may lead to inaccurate associations between migraine and diet itself. Consequently, it is also challenging to draw meaningful conclusions about the efficacy of the intervention. These circumstances may lead to significant study bias, making the results difficult to apply to clinical practise. 

Regarding the elimination diets, neither Alpay et al. [18] or Özön et al. [20,21] presented a clear approach on how to detect the foods that were eliminated or restricted. Without a clear approach, it may be challenging to replicate the study, build on its findings, or interpret the results accurately.

Alpay et al.’s study [18] was the only one to have patients and physicians be blinded to the IgG test results compared to the others. Arab et al. [17] was unable to blind the participants and researchers, except the neurologist responsible for conducting all the clinical assessments. As for Lovati et al. [16], the pilot study was non-double-blinded and Bunner et al. [19] could not blind participants or instructors. The other studies did not address blinding. This situation could, once again, introduce bias into the studies. The intervention group may show more favourable outcomes due to the assumption of receiving better treatment than a control group. Adherence to the dietary regimens is also a crucial limitation, as monitoring adherence is challenging, and non-compliance can affect the trial’s overall outcome. To ensure better diet compliance, interventions must be properly instructed and guided regarding the dietetic approach’s nutritional facts and key points. This, however, may be time-consuming to carry out, especially when we need larger sample sizes in future studies. Another general concern is the short duration of the diets themselves and the subjective measures of the duration, frequency, and pain intensity of migraine attacks. The different parameters are often self-reported, introducing subjectivity. Participants’ perceptions of their diets’ effects on migraines can also be influenced by expectations or placebo effects, potentially giving biassed results. For instance, this is noticeable in the study by Alpay et al. [18], where participants receiving the provocation diet demonstrated a non-significant, yet notable, improvement in the migraine parameters. Ergo, a decrease was observed in mean attack count, mean headache days per month, and mean total medication intake, contrary to the expected increase. Variability in reactions to food and what triggers a migraine attack is also an individual case. 

One author used the Newcastle–Ottawa Quality Assessment Scale to evaluate the quality of the eight studies. The study by Arab et al. [17] was rated highest, demonstrating the strength of a controlled environment. The studies by Bunner et al. [19] and Alpay et al. [18], which received the next highest scores, also highlight the value of a randomised crossover design. The studies by Özön et al. [20,21] and Ameghino et al. [22] were rated with the same scores; however, a randomised crossover or controlled study design ensures a high level of internal validity. In contrast, the studies by Lovati et al. [16] and Bongiovanni et al. [15] were rated lower in quality, emphasising the importance of control groups and robust study design. The limitations of having only one author assess publications using this tool include subjectivity and increased bias. The rating may reflect the personal biases and interpretations of the single reviewer, leading to less objectivity in scoring and lack of reliability.

Lastly, the studies were screened by only one reader, which can significantly impact the quality and reliability of this review.

## 5. Conclusions

Some studies have demonstrated that certain diets may improve attack duration, frequency, severity, and medication use in migraine patients. At present, the overall evidence for recommending certain diets for migraine is weak. There is a high need for further large double-blinded RCTs, which can demonstrate if specific diets can be used with confidence in the armamentarium of migraine treatment.

## Figures and Tables

**Table 1:** Eligibility criteria for study inclusion using PICOS.

	Inclusion Criteria	Exclusion Criteria
**Population (P)**	Adult patients (≥18 years)	Paediatric patients (≤18 years)
**Intervention (I)**	Food, dietary, and nutritional	Supplements, vitamins, medication
**Comparison (C)**	Interventions and placebo/control	Macronutrients, caloric intake, or weight/BMI
**Outcome (O)**	Diets as triggering factors or treatment/prevention of migraine	-
**Study design (S)**	Retrospective or prospective observational studies, RCTs, and non-RCTs	Conference papers, guidelines, opinions, editorials, letters, case reports, book chapters, or comments
**Language**	English	Non-English
**Other**	A diagnosis of migraine must meet the criteria outlines in any iteration of the ICHD	Studies with less than 10 patients If data could not be extracted properly Studies reporting results on overlapping cohorts

**Table 2:** Scores for the Newcastle–Ottawa Quality Assessment Scale (NOS) for cohort studies.

Author, Year	Diet Intervention	NOS Score
Arab, A. et al., 2022 [17]	DASH diet	7
Bunner, A. E. et al., 2014 [19]	Low-fat vegan diet and elimination diet	4
Alpay, K. et al., 2010 [18]	Elimination diet	4
Özön, A. Ö. et al., 2018 [20]	Elimination diet	4
Özön, A. Ö. et al., 2021 [21]	Elimination diet	4
Ameghino, L. et al., 2019 [22]	Gluten-free diet	4
Lovati, C. et al., 2022 [16]	Ketogenic diet or low-carb diet	3
Bongiovanni, D. et al., 2021 [15]	Ketogenic diet	2

## Supplementary Materials

The following supporting information can be downloaded at: https://www.mdpi.com/article/10.3390/nu16193415/s1, Table S1: Studies with diet intervention and food item triggers.
