# Supplementary material for: The Evidence for Diet as a Treatment in Migraine—A Review
Source: Nutrients. 2024 Oct 9;16(19):3415. doi: 10.3390/nu16193415 (PMC11478386; doi:10.3390/nu16193415)
Supplement: Supplementary file 1 [file nutrients-16-03415-s001.zip › nutrients-3199643-supplementary.pdf]

Table S1: Studies with diet interventions and food item triggers

Author, year	Study design	ICHD	Sample size	Gender	Types of migraine	Method/Diet intervention	Results	Conclusion
Alpay, K. et al., 2010 [18]	Clinical double-blind, randomised cross-over trial	2	(n = 30)	F: (n = 28)<br>M: (n = 2)	MoA (n = 30)	Randomised to a 6-week diet including (provocation diet) or excluding (elimination diet) specific foods with high concentration of IgG antibodies with a 2-week wash-out period before second evaluation period.	Patients with positive IgG test results: Spices (n = 27), seeds and nuts (n = 24), seafood (n = 24), vegetables (n = 21), cheese (n = 20), fruits (n = 20), sugar products (n = 20).<br><br>Mean attack duration for baseline: 11.39±5.6h, after provocation diet: 12.53±6.7h and after elimination diet: 12.53±6.7h (*p*<0.8).<br>Mean migraine attack counts for baseline: 8.97±4.4, after provocation diet: 8.13±4.6 and after elimination diet: 6.17±3.8 (*p*<0.001*).<br>Mean headache days per month for baseline: 10.5±4.4, after provocation diet: 10.20±5.5 and after elimination diet: 7.47±3.7 (*p*<0.001*).<br>Mean median headache intensity (VAS) for baseline: 6.02±1.6, after provocation diet: 6.07±1.6 and after elimination diet: 6.07±1.6 (*p*<0.5).<br>Mean total medication intake for baseline: 11.37±7.4 tablets, after provocation diet: 10.57±7.7 tablets and after elimination diet: 7.77±5.7 tablets (*p*<0.01*).	The frequency of migraine attacks was effectively reduced based on the IgG antibody diet elimination. The small sample size requires caution while translating the results into practice.
Bunner, A. E. et al., 2014 [19]	Interventional, randomised cross-over trial	2	(n = 42)	F: (n = 39)	NA	Randomised to a 16-week dietary instruction or placebo supplement with a 4-week wash-out period before second evaluation period.<br><br>A low-fat vegan diet was prescribed for 4 weeks, after which an elimination diet was used to enable patients to identify possible trigger foods. During the elimination diet period, patients were asked to continue the low-fat vegan diet and to eliminate food chosen based on previous studies (wheat, rye, barley, corn, soybeans, chickpeas, peanuts, all citrus fruits, bananas, apples, tomatoes, eggplants, peppers, potatoes, onions, garlic, sweet potatoes, yams, celery, all animal products, all nuts and seeds, chocolate, sugar, coffee, tea and alcohol). Patients were asked to adhere to the elimination diet until no further improvement was noted or until the period midpoint, after which they were asked to reintroduce the omitted foods one at a time, starting with foods least likely to be a trigger, every 48h.<br>The placebo was a capsule containing 10 μg alpha-linolenic acid and 10 μg vitamin E.	Diet intervention:<br>Mean attack duration for baseline: 6.1±4.1h, after diet intervention: 4.8±3.6h (*p*<0.01*).<br>Mean migraine attack counts per week for baseline: 2.1±1.4, after diet intervention: 1.7±1.9.<br>Mean headache days per week for baseline: 2±1.4, after diet intervention: 1.7±1.6. (*p*<0.05*).<br>Mean pain intensity (VAS) for baseline: 6±2.7, after diet intervention: 3.6±3 (*p*<0.0001*)<br>Mean headache intensity (VAS) for baseline: 4.3±1.8, after diet intervention: 3.1±1.8 (*p*<0.001*).<br>Mean percentage of medicated headaches for baseline: 65.2±31.8% and after diet intervention: 41.3±31.3 (*p*<0.001*)<br><br>Placebo supplement:<br>Mean attack duration for baseline: 4,5±3.2h, after supplement period: 4.4±3.6h.<br>Mean migraine attack counts per week for baseline: 2.1±2, after supplement period: 1.8±2.2 (*p*<0.03*).<br>Mean headache days per week for baseline: 1.9±1.6, after supplement period: 1.6±1.6 (*p*<0.03*)<br>Mean pain intensity (VAS) for baseline: 4.7±2.8, after supplement period: 4.1±2.8.<br>Mean headache intensity (VAS) for baseline: 3.6±1.9, after supplement period: 3.2±2.<br>Mean percentage of medicated headaches for baseline: 50.1±32.3 and after supplement period: 46.1±33.6.	The frequency, intensity and duration of migraine attacks was effectively reduced based on a low-fat vegan diet along with an elimination diet.<br>Further studies are needed to confirm usefulness of the diet and isolate dietary triggers.
Özön, A. Ö. et al., 2018 [20]	Interventional and randomised	2	(n = 50)	Group 1<br>F: (n = 21)<br>M: (n = 4)<br><br>Group 2<br>F: (n = 20)<br>M: (n = 5)	MoA (n = 50)	Patients were randomly divided into two groups. The identified triggering foods were excluded from the diet of the patients in groups 1 and 2. The elimination diet continued for 4 months. Diet restriction was relaxed in group 1 after the second month but continued in group 2.	Group 1:<br>Mean attack duration for baseline: 29.44±21.8h, after second month: 22.2±15.2h (*p*<0.05*) and after fourth month: 28.96±20h (*p*<0.8).<br>Mean migraine attacks per month for baseline: 6.08±1.7, after second month: 4.84±1.9 (*p*<0.02*) and after fourth month: 5.96±1.7 (*p*<0.5).<br>Mean pain intensity (VAS) for baseline: 89.80±5.7, after second month: 72.89±10.9 (*p*<0.01*) and after fourth month: 86.80±9.6 (*p*<0.2).<br><br>Group 2:<br>Mean attack duration for baseline: 30.56±22.3h, after second month: 23.52±18.1h (*p*<0.04*) and after fourth month: 22.88±18.4h (*p*<0.03*).<br>Mean migraine attacks per month for baseline: 5.96±1.7, after second month: 4.68±1.9 (*p*<0.02*) and after fourth month: 4.64±1.8 (*p*<0.01*).<br>Mean pain intensity (VAS) for baseline: 90.20±8.5, after second month: 72.20±19.9 (*p*<0.01*) and after fourth month: 71.40±20.1 (*p*<0.01*).<br><br>Patients affected by the food items: Wheat (n = 13/14); Orange (n = 10/12); Egg (n = 11/9); Nescafe (n = 9/8); Cheese (n = 9/7); Chocolate (n = 7/7); Milk (n = 7/8); Sujuk (n = 6/7); Sugar, beet (n = 6/6); Red meat (n = 6/5); Pickle (n = 6/5); Alcohol (n = 5/5); Mushroom (n = 4/5); Corn (n = 3/4); Tea (n = 3/3); Onion (n = 2/1); Garlic (n = 1/1) for group 1/group 2, respectively.	Migraine-triggering foods identified by migraine patients was an effective and reliable method to reduce migraine symptoms when restricting the intake of these. The intervention contributed to prevention and minimisation of migraine attacks.

Table S1: Continued

Author, year	Study design	ICHD	Sample size	Gender	Types of migraine	Method/Intervention	Results	Conclusion
Ameghino, L. et al., 2019 [22]	Interventional	3	(n = 866)<br><br>Total number of patients with migraine: (n = 414 of sample size)	F: (n = 394 of total number of patients with migraine)<br>M: (n = 20 of total number of patients with migraine)	MoA (n = 281)<br>MWA (n = 133)<br>EM (n = 242 of MoA and 114 of MWA)	Analysing an online survey about the characteristics of headache and its response to a 2-month gluten free diet in migraine patients with celiac disease.	Baseline duration for patients with MoA: 0.5-4h (n = 115), 4-24h (n = 114) and >24h (n = 52) and for patients with MWA: 0.5-4h (n = 65), 4-24h (n = 41) and >24h (n = 27).<br><br>Baseline frequency for patients with MoA: 1-4d (n = 121), 5-9d (n = 87), 10-14d (n = 34) and >15d (n = 34) and for patients with MWA: 1-4d (n = 52), 5-9d (n = 38), 10-14d (n = 24) and >15d (n = 17).<br><br>Frequency for patients with MoA: improvement was seen in 52,4%, a decrease was seen in 10% and 37,6% were without changes and for patients with MWA: improvement was seen in 46.1%, a decrease was seen in 12.4% and 41.5% were without changes (p=0.02*).<br>Pain intensity (NRS) for patients with MoA: improvement was seen in 49.6%, a decrease was seen in 11% and 39.4% were without changes and for patients with MWA: improvement was seen in 46.2%, a decrease was seen in 10.8% and 43% were without changes (p=0.013*).	Results suggest that a strict compliance to a gluten free diet could improve headaches in celiac patients with headache, even in those without GI symptoms.
Bongiovanni, D. et al., 2021 [15]	Clinical, interventional trial	3	(n = 38)	F: (n = 36)<br>M: (n = 2)	CM (n = 38)	3-month ketogenic diet (KD) followed by a transition phase (1 month) of reintegration of carbohydrate and a maintenance phase (2 months). There was also a monthly follow-up for 3 months.<br>The diet set a limit of a maximum 30g carbohydrate, 1.3-1.5g protein per kg of bodyweight and 35-80% of lipids of the total amount of calories a day. Additionally, a mineral and vitamin supplement was prescribed.	Median duration of migraine attacks for baseline: 24h and after ketogenic diet: 5.5h (p<0.002*).<br>Median headache days per month for baseline: 30 and after ketogenic diet: 7.5 (p<0.001*).<br>Median pain intensity for baseline: at maximum (severe) for 83% and after ketogenic diet: improved (mild) for 55% of them (p<0.003*).<br>Median number of drugs taken a month for baseline: 30 doses and after ketogenic diet: 6 doses (p<0.003*).<br><br>Two patients had no change in the duration.<br>Four patients failed to see any benefits from the diet.	The 3-month KD showed a significant reduction in duration, frequency, pain intensity of migraine attacks and the number of analgesics taken per month.
Özön, A. Ö. et al., 2021 [21]	Interventional	3	(n =31)	F: (n = 23)<br>M: (n = 8)	MoA (n = 31)	2-month elimination diet. The identified triggering foods were excluded from the diet of the elderly patients.	Seventeen foods were determined as a trigger, of which all of them were excluded from the diets: Wheat (n = 15), egg (n = 14), cheese (n = 12), Nescafé (n = 11) and milk (n = 11) were determined the most triggering foods. Other foods were chocolate (n = 9), alcohol (n = 9), sujuk (n = 7), tea (n = 6), red meat (n = 6), onion (n = 6), pickle (n = 5), orange (n = 4), oat (n = 3), grape (n = 3), garlic (n = 2) and sesame (n = 2).<br><br>Mean attack duration for baseline: 32.65±19.38h and after elimination diet: 18.74±13.99h (p<0.001*).<br>Mean migraine attack counts for baseline: 5.74±1.55 and after elimination diet: 4.16±2.02 (p<0.002*).<br>Mean pain severity (VAS) for baseline: 82.26±9.9 and after elimination diet: 62.26±22.2 (p<0.001*).<br>Mean number of used analgesics for baseline: 4.94±1.34 and after elimination diet: 2.74±1.98 (p<0.001*)<br>Mean number of used triptans for baseline: 1.65±1.83 and after elimination diet: 0.87±1.45 (p<0.002*)	After dieting in the second month, frequency, attack duration, pain severity and analgesic and triptan counts were significantly lower than pre-treatment levels.<br><br>Some foods may trigger migraine attacks in elderly migraine patients and an elimination diet may be an effective and reliable treatment in reducing the clinical symptoms and number of analgesics and triptans used in migraine attacks.
Arab, A. et al., 2022 [17]	Clinical Interventional, randomised controlled trial	3	(n = 102)	F: (n = 102)	MWA (n = 33)<br>EM (n = 82)	Randomised to a 12-week “Dietary Approaches to Stop Hypertension” diet (DASH diet) or usual diet (control).<br>The 7-day menu cycle provided protein, fats, and carbohydrate of 15-20%, 25-30% and 55-60% of the total daily energy, respectively.<br>The DASH diet contained grains (7 servings/d), vegetables (5 servings/d), fruits (5 servings/d), dairy products (3 servings/d), and meats, poultry, and fish (3 servings/d). It also contained nuts, seeds, and legumes (1 servings/d) and fats and oils (7 servings/d).<br>The DASH diet has higher amounts of vegetables, fruits, whole grains, low-fat dairy, nuts and beans compared to the control diet. The diet also has lower amounts of red meat, sweets, refined grains, saturated fats, and cholesterol compared to the control diet.<br>Both groups were instructed to limit or eliminate consuming cheese, chocolate, citrus fruits, processed meat, hot dogs, ham, fatty and fried foods, and canned foods.	Three in the DASH diet group and three in the control group were lost to follow-up.<br>DASH:<br>Mean attack duration for baseline: 1.11±0.12d and after DASH diet: 0.52±0.05d (p<0.001*)<br>Mean migraine attack counts per month for baseline: 8.15±0.78 and after DASH diet: 5.15±0.48 (p<0.001*).<br>Mean pain intensity (VAS) for baseline: 7.78±0.21 and after DASH diet: 6.01±0.21 (p<0.001*)<br><br>Control:<br>Mean attack duration for baseline: 1.04±0.12d and after usual diet: 0.71±0.08d (p<0.01*).<br>Mean migraine attack counts per month for baseline: 8.98±1.09 and after usual diet: 7.57±0.97 (p=0.113).<br>Mean pain intensity (VAS) for baseline: 7.5±0.3 and after usual diet: 6.9±0.25 (p<0.02*).	The study included hypertensive patients and did not assess women’s menstrual cycle status. This makes it challenging to attribute any positive results on migraine directly. The 12-week DASH diet may, however, still improve the clinical symptoms such as frequency, duration, and severity of migraine in women compared to a control diet.

Table S1: Continued

Author, year	Study design	ICHD	Sample size	Gender	Types of migraine	Method/Intervention	Results	Conclusion
Lovati, C. et al., 2022 [16]	Non-double-blind, nonrandomised, pilot study	3	(n = 53)<br><br>Study 1: (n = 21 of sample size)<br><br>Study 2: (n = 32 of sample size)	Study 1<br>KD group<br>F: (n = 11)<br>M: (n = 2)<br><br>LCD group<br>F: (n = 8)<br><br>Study 2<br>KD group<br>F: (n = 24)<br>M: (n = 2)<br><br>LCD group<br>F: (n = 5)<br>M: (n = 1)	CM (n = 37)	In study 1, patients followed either a ketogenic diet (KD, n = 13) or low carb diet (LCD, n = 8) regimen for 3 consecutive weeks. Regimens: KD group, normocaloric or hypocaloric Modified Atkins Diet tailored on the patient enriched with medium-chain triglyceride oils and alkaline inducers; LCD group, tailored diet with a reduced content (<40%) of carbohydrates.<br><br>In study 2, 26 were included in the KD group and 6 in the LCD group.	Study 1:<br>KD – Mean headache days per month for baseline: 19.1±6.5 and after ketogenic diet: 12.3±9.2 ($p<0.05$*).<br>Mean pain intensity (NRS) for baseline: 7.8±0.7 and after ketogenic diet: 5.7±2 ($p=0.01$*).<br>Mean medication intake per month for baseline: 24.9±18.7 doses and after ketogenic diet: 11.5±10.7 doses ($p=0.05$*).<br><br>LCD – Mean headache days per month for baseline: 21±6.5 and after low-carb diet: 18.6±7.8.<br>Mean pain intensity (NRS) for baseline: 7±1.4 and after low-carb diet: 6.4±1.5.<br>Mean medication intake per month for baseline: 27.8±14 doses and after low-carb diet: 23.8±11.7 doses.<br><br>Study 2:<br>Only 15/26 of the KD group and 1/6 of the LCD group were responders.<br>KD – Mean headache days per month for baseline: 14.5 and after ketogenic diet: 10.5 ($p=0.1$).<br>Among the 15 responders, it decreased from 11.6 to 4.9 ($p<0.001$*).	The pilot study suggests that a ketogenic diet might be useful and worth to be carried out in refractory migraine.

Abbreviations: ICHD = International Classification of Headache Disorders; F = Females; M = Males; MoA = Migraine without aura; MWA = Migraine with aura; EM = Episodic migraine; CM = Chronic migraine; NRS = Numerical Rating Scale; VRS = Visual Analog Scale;
* significant
